# A Systematic Review and Meta‐Analysis of the Efficacy and Safety of Propranolol Versus Other Drugs in the Treatment of Infantile Hemangioma

**DOI:** 10.1111/jocd.70750

**Published:** 2026-02-17

**Authors:** Jiahua Hu, Lisha Pan, Hong Kong, Jiaqi Lou

**Affiliations:** ^1^ Department of Pediatric Surgery Huzhou Maternity & Child Health Care Hospital Huzhou City China; ^2^ Huzhou Central Hospital Fifth School of Clinical Medicine of Zhejiang Chinese Medical University Huzhou City China; ^3^ Ningbo College of Health Sciences Ningbo China; ^4^ Burn Department Ningbo No. 2 Hospital, Wenzhou Medical University Ningbo China

**Keywords:** atenolol, efficacy, infantile hemangioma, meta‐analysis, propranolol, safety, systematic review

## Abstract

**Objective:**

This study aimed to systematically evaluate and compare the efficacy and safety of propranolol versus atenolol, corticosteroids, timolol, and other therapies in the treatment of infantile hemangioma (IH) through a meta‐analysis, thereby providing evidence‐based guidance for clinical practice.

**Methods:**

A comprehensive literature search was conducted across PubMed, Cochrane Library, EMBASE, Web of Science, CNKI, and Wanfang databases from inception to December 2025. The protocol was prospectively registered with PROSPERO (CRD420261294316). Randomized controlled trials (RCTs) or clinical controlled trials (CCTs) comparing oral propranolol with other active drugs in IH patients aged ≤ 12 years were included. Primary outcomes were overall response rate (≥ 50% reduction), complete remission rate, and incidence of adverse events. Two reviewers independently performed study selection, data extraction, and quality assessment using the Cochrane RoB 2.0 tool and Newcastle‐Ottawa Scale. Pooled odds ratios (ORs) with 95% confidence intervals (CIs) were calculated using R software. Heterogeneity was assessed using the *I* ^2^ statistic.

**Results:**

Eight studies involving 900 patients (propranolol: 464; control: 436) were included. Meta‐analysis revealed no statistically significant difference in overall response rate between propranolol and control groups (pooled OR = 1.29, 95% CI: 0.80–2.09, *p* = 0.30). However, propranolol demonstrated a significantly higher complete remission rate (OR = 1.35, 95% CI: 1.01–1.82, *p* = 0.045). Subgroup analyses by control drug type (atenolol, corticosteroids, timolol, combination therapy) showed no significant differences in efficacy (all *p* > 0.05). Safety analysis indicated no significant difference in adverse event incidence between groups (OR = 0.76, 95% CI: 0.38–1.55, *p* = 0.45), albeit with moderate heterogeneity (*I* ^2^ = 56%). Heterogeneity was low for efficacy outcomes (*I* ^2^ = 0%). Funnel plot symmetry and a non‐significant Egger's test suggested a low risk of publication bias.

**Conclusion:**

Propranolol offers a statistically significant advantage in achieving complete remission of infantile hemangioma compared to other active agents, while maintaining comparable overall response rates and a similar overall safety profile. These findings support propranolol as a first‐line therapy when complete lesion resolution is the primary goal. Atenolol represents an effective alternative, particularly for patients with specific tolerability concerns, underscoring the need for individualized treatment selection.

## 1 Background

Infantile hemangioma (IH), the most common benign vascular tumor of infancy, represents a significant clinical entity in pediatric dermatology and vascular anomalies. Its reported incidence ranges from 4% to 10% in full‐term infants and can be as high as 30% in preterm neonates, with a notable female predominance of approximately 3:1 [1, 2]. The classic natural history of IH is characterized by a distinct triphasic trajectory: a initial proliferative phase of rapid growth typically beginning in the first few weeks of life, a subsequent stabilization or plateau phase, and a final, prolonged involution phase that can extend over several years [3]. While the majority of IHs follow this benign, self‐limiting course, a substantial subset—estimated at 10%–15% [4]—are considered problematic due to their potential for causing severe complications. These include ulceration with attendant pain and risk of infection, bleeding, functional impairment (such as visual axis obstruction, airway compromise, or feeding difficulties [5]), and permanent cosmetic disfigurement with associated psychosocial sequelae. The unpredictability of growth and the risk of sequelae in certain high‐risk locations (e.g., periorbital, parotid, perineal, segmental facial lesions [6]) have historically driven the need for active therapeutic intervention.

The therapeutic landscape for IH has undergone a paradigm shift over the past 15 years. Prior to 2008 [7], systemic and intralesional corticosteroids were the mainstay of pharmacotherapy for complicated IH, despite a side‐effect profile that included cushingoid features, growth retardation, hypertension, and immunosuppression [7, 8]. The serendipitous discovery by Léauté‐Labrèze et al. [9] of the dramatic efficacy of the non‐selective β‐adrenergic receptor antagonist propranolol revolutionized management, establishing it as the de facto first‐line systemic therapy. Propranolol's mechanism of action, though not fully elucidated, is believed to be multifactorial, encompassing initial vasoconstriction via inhibition of nitric oxide release, subsequent blockade of angiogenesis through downregulation of key pro‐angiogenic factors like VEGF and bFGF [10, 11], and induction of apoptosis in hemangioma‐derived endothelial cells. Despite its high efficacy, propranolol is not without limitations. Its non‐selective nature is associated with a spectrum of potential adverse effects, including bradycardia, hypotension, hypoglycemia, bronchospasm, sleep disturbances, and gastrointestinal upset, necessitating pretreatment evaluation and often dose titration [12]. This safety profile has spurred investigation into alternative beta‐blockers. Atenolol, a hydrophilic, selective β1‐antagonist, offers theoretical advantages of fewer central nervous system and pulmonary side effects due to its cardioselectivity and poor blood–brain barrier penetration [13], positioning it as a potential alternative, especially for patients with asthma or concerns about sleep [14, 15]. Topical timolol, a non‐selective blocker, has emerged as a valuable option for superficial, low‐risk lesions [15, 16]. Meanwhile, corticosteroids have been relegated to a secondary or adjunctive role [16, 17, 18].

Consequently, a critical and evolving debate in the field centers on the comparative efficacy and safety profile of propranolol versus other active agents, particularly atenolol [19, 20, 21]. While numerous primary studies and several meta‐analyses have been conducted, consensus remains elusive, with heterogeneity among existing studies [22, 23, 24] in terms of design, patient populations, outcome definitions, and treatment protocols. The majority have focused on synthesizing data from single‐arm studies or have employed network meta‐analyses to indirectly compare multiple agents [22, 23, 24]. A direct, pairwise meta‐analysis focusing exclusively on head‐to‐head comparative studies (RCTs and CCTs) of propranolol versus other active drugs is less common. This approach, by design, excludes single‐arm efficacy data, which limits its ability to quantify the absolute efficacy of propranolol. However, it offers a distinct advantage: it minimizes confounding by time and population differences inherent in comparing separate single‐arm series, thereby providing a more rigorous and direct estimate of relative efficacy and safety. Therefore, this study aims to perform a systematic review and pairwise meta‐analysis to directly and quantitatively compare the clinical efficacy and safety of propranolol against atenolol and other active comparators (corticosteroids, timolol) in the treatment of infantile hemangioma, addressing a specific gap in the comparative evidence landscape.

## 2 Materials and Methods

### 2.1 Study Design and Registration

This investigation was conducted as a systematic review and meta‐analysis of comparative clinical studies. The protocol was designed a priori in accordance with the Preferred Reporting Items for Systematic Reviews and Meta‐Analyses (PRISMA) 2020 statement to ensure methodological rigor and transparent reporting [24]. The review protocol was prospectively registered on the International Prospective Register of Systematic Reviews (PROSPERO) (Registration number: CRD420261294316).

### 2.2 Literature Search Strategy

A comprehensive and systematic literature search was executed to identify all relevant published studies. The electronic databases searched included PubMed/MEDLINE, the Cochrane Central Register of Controlled Trials (CENTRAL), EMBASE, Web of Science Core Collection, China National Knowledge Infrastructure (CNKI), Wanfang Data, and VIP Chinese Journal Database. Chinese‐language databases were included because infantile hemangioma is a condition of high clinical interest in China, and these databases index a substantial body of rigorous clinical research not always captured in international databases. Their inclusion aims to minimize “database bias” and provide a more comprehensive global evidence base [18]. The search timeframe spanned from the inception of each database through December 1, 2025. No language restrictions were applied initially during the database search.

The search strategy employed a combination of controlled vocabulary (e.g., MeSH terms in PubMed, EMTREE in EMBASE) and free‐text keywords to maximize sensitivity [25]. Key search concepts included: (1) the population: “infantile hemangioma” OR “capillary hemangioma” OR “strawberry hemangioma”; (2) the interventions: “propranolol” OR “beta‐blocker” OR “β‐blocker”; (3) the comparators: “atenolol” OR “timolol” OR “corticosteroids” OR “steroids” OR “prednisolone”; and (4) the study design: “randomized controlled trial” OR “controlled clinical trial”. These concepts were combined using the Boolean operator “AND”. The specific search syntax was adapted for the unique features of each database. For instance, a representative PubMed search strategy is provided below: (“infantile hemangioma” [MeSH Terms] OR “infantile hemangioma” [Title/Abstract] OR “capillary hemangioma” [Title/Abstract]) AND (“propranolol” [MeSH Terms] OR “propranolol” [Title/Abstract] OR “beta blocker” [Title/Abstract]) AND (“atenolol” [MeSH Terms] OR “atenolol” [Title/Abstract] OR “timolol” [Title/Abstract] OR “corticosteroids” [MeSH Terms] OR “steroids” [Title/Abstract]) AND (“randomized controlled trial” [Publication Type] OR “controlled clinical trial” [Publication Type] OR “randomized” [Title/Abstract] OR “randomly” [Title/Abstract]). Additionally, the reference lists of all included studies and relevant systematic reviews were manually screened to identify any potentially eligible articles not captured by the electronic search.

### 2.3 Study Selection and Eligibility Criteria

The study selection process was performed independently by two reviewers using the web‐based systematic review software Covidence to manage records and resolve conflicts. The process consisted of two sequential screening phases [26, 27]:

Title and abstract screening: Duplicate records were removed automatically and manually. The remaining unique records were screened based on their titles and abstracts against the pre‐defined eligibility criteria.

Full‐text review: The full texts of all records deemed potentially eligible or with uncertain eligibility from the first phase were retrieved and assessed in detail.

Any discrepancies between reviewers at either stage were resolved through discussion or, if necessary, by consultation with a third senior reviewer.

The eligibility criteria were as follows:

Population: Infants and children (aged ≤ 12 years) with a clinically or pathologically diagnosed infantile hemangioma (IH), regardless of lesion size, location, or subtype. The upper age limit of 12 years was set to encompass the vast majority of the active treatment and involution phases of IH, while also including studies that may have enrolled older children with late‐presenting or persistent lesions requiring therapy.

Intervention: Oral propranolol, administered as monotherapy or as part of a combination regimen.

Comparator: An active control treatment, including but not limited to oral atenolol, oral or intralesional corticosteroids, topical timolol, or a combination of different active drugs.

Outcomes: Studies had to report data on at least one of the following primary outcomes: (a) Overall treatment response, which we defined for the purpose of this review as a reported “good” or “excellent” response, or a quantitative reduction (typically ≥ 50%) in lesion size, volume, or a composite score. We documented the specific criteria used in each individual study; (b) Complete remission, defined as the complete or near‐complete resolution of the hemangioma; (c) Incidence of adverse events.

Study design: Randomized controlled trials (RCTs) or non‐randomized controlled clinical trials (CCTs) with a parallel‐group design.

Studies were excluded if they were: case reports, case series, editorials, reviews, conference abstracts without full data; in vitro or animal studies; lacking a relevant control group (e.g., placebo, observation, or a different active drug as defined above); or published in languages other than English or Chinese. While the initial search was language‐unrestricted, practical constraints in ensuring accurate data extraction and quality assessment limited full‐text inclusion to English and Chinese studies [28].

### 2.4 Data Extraction

A standardized, pilot‐tested data extraction form was developed in Microsoft Excel. Two reviewers independently extracted data from each included study. The extracted information included:

Study characteristics: first author, publication year, journal, country/region, study design (RCT/CCT), study duration, follow‐up period.

Participant characteristics: total sample size, number of participants in each group, age (mean/median and range), gender distribution, baseline characteristics of the IH (location, size, subtype if reported).

Intervention details: drug name, dosage (e.g., mg/kg/day), administration route, treatment duration, any concomitant therapies.

Outcome data: For dichotomous outcomes (response, remission, adverse events), the number of events and the total number of participants in each group were extracted. For the “overall response” outcome, the exact definition and assessment method used in the study (e.g., “≥ 50% reduction in size by visual inspection”, “improvement score ≥ 3 on a 4‐point scale”) were recorded verbatim. For continuous outcomes, means and standard deviations were sought.

Other data: Funding sources, author declarations of interest, and key conclusions.

Any discrepancies in extracted data were cross‐checked against the original publication and resolved by consensus.

### 2.5 Assessment of Risk of Bias

The methodological quality and risk of bias of the included studies were assessed independently by two reviewers. For RCTs, the revised Cochrane Risk of Bias tool for randomized trials (RoB 2.0) was employed as per the official Cochrane guidance [29]. This tool evaluates five domains: (1) bias arising from the randomization process, (2) bias due to deviations from intended interventions, (3) bias due to missing outcome data, (4) bias in measurement of the outcome, and (5) bias in selection of the reported result. Each domain was judged as “Low risk,” “Some concerns,” or “High risk,” leading to an overall risk of bias judgment for each study.

For CCTs, the Newcastle‐Ottawa Scale (NOS) [30], adapted for non‐randomized studies, was used. The NOS assesses studies on three broad perspectives: (1) the selection of the study groups (0–4 stars), (2) the comparability of the groups (0–2 stars), and (3) the ascertainment of the outcome of interest (0–3 stars). A total score of ≥ 7 stars was considered indicative of high quality, 4–6 stars as moderate quality, and ≤ 3 stars as low quality.

### 2.6 Statistical Analysis

All statistical analyses were performed using R statistical software (version 4.3.1; R Foundation for Statistical Computing). The primary meta‐analyses were conducted using the metafor package (version 4.0‐0) [31]. For dichotomous outcome measures (treatment response, complete remission, adverse events), treatment effects were expressed as Odds Ratios (ORs) with corresponding 95% Confidence Intervals (CIs). An OR > 1 favored the propranolol group for efficacy outcomes, while an OR < 1 favored the propranolol group for safety (adverse events), indicating a lower odds of an adverse event.

Heterogeneity across studies was assessed using the *I* ^2^ statistic and the Cochrane's *Q* test [32]. The *I* ^2^ statistic quantifies the proportion of total variation in study estimates that is due to heterogeneity rather than chance, with values of 25%, 50%, and 75% typically considered indicative of low, moderate, and high heterogeneity, respectively. The significance level for the *Q* test was set at *p* < 0.10. Based on the heterogeneity assessment, the choice of the pooling model was made:

A fixed‐effect model (Mantel–Haenszel method) was used when substantial heterogeneity was not present (*I* ^2^ ≤ 50% and *p* ≥ 0.10 for the *Q* test).

A random‐effects model (DerSimonian and Laird method) [33, 34] was applied when significant heterogeneity was detected (*I* ^2^ > 50% or *p* < 0.10 for the *Q* test), as it accounts for variability both within and between studies.

To address potential bias arising from clinical heterogeneity, particularly variations in the definition of “overall response” across studies, we performed a secondary analysis using a random‐effects model irrespective of *I* ^2^ for this outcome and conducted a sensitivity analysis by excluding studies whose response criteria deviated most from the common ≥ 50% reduction threshold. These methods help assess the robustness of our findings against variability in study design and outcome measurement.

Subgroup analyses were pre‐specified to explore potential sources of heterogeneity and to examine the consistency of effects across different clinical contexts. Subgroups were defined by: (1) Type of Control Drug: Atenolol versus Corticosteroids versus Timolol versus Combination therapy; (2) Geographic Region: Asia versus Europe versus Americas; (3) Study Quality/Risk of Bias: High quality (RoB 2.0: Low risk/NOS: ≥ 7 stars) versus Moderate/Low quality; (4) Differences between subgroups were tested formally using meta‐regression (for categorical subgroups with sufficient studies) or by inspecting the overlap of confidence intervals.

Sensitivity analyses were conducted to test the robustness of the primary findings. The primary method was the leave‐one‐out analysis, where the meta‐analysis was repeated iteratively, each time omitting a single study to assess whether any individual study exerted a disproportionate influence on the pooled estimate [35].

Assessment of publication bias was performed visually by inspecting funnel plots (plotting the standard error against the log odds ratio) for asymmetry. This was supplemented by Egger's linear regression test, a statistical test for funnel plot asymmetry where a *p*‐value < 0.05 was considered suggestive of potential publication bias [36].

## 3 Results

### 3.1 Study Selection Process and Characteristics of Included Studies

**FIGURE 1:**
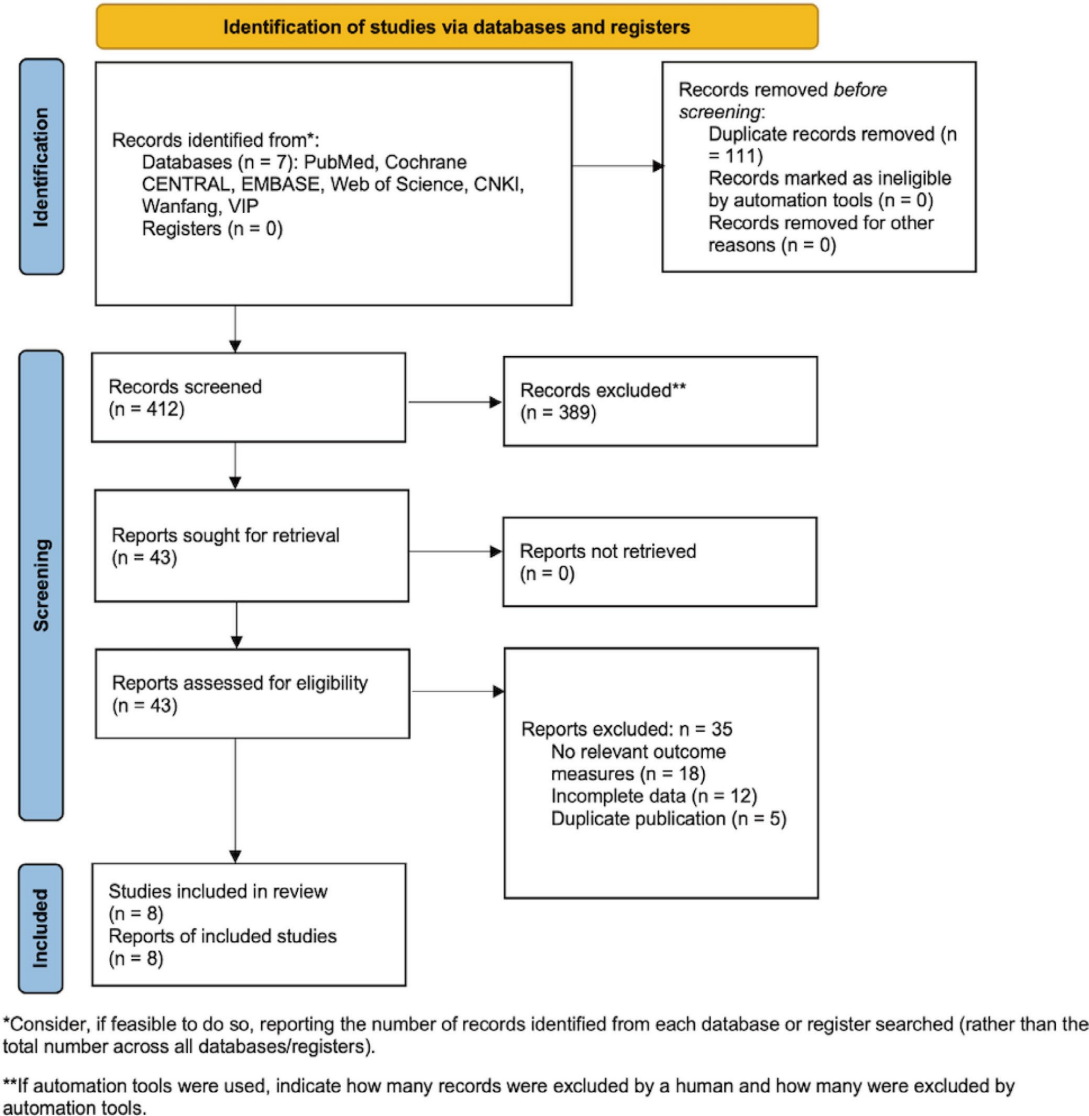
PRISMA flow diagram of study selection for the systematic review and meta‐analysis of propranolol versus other drugs for infantile hemangioma. This diagram illustrates the sequential process of study identification, screening, eligibility assessment, and inclusion according to the Preferred Reporting Items for Systematic Reviews and Meta‐Analyses (PRISMA) guidelines. The numbers of records at each stage are shown, along with specific reasons for exclusion. A total of 523 records were identified through database searching. After duplicate removal and screening of titles/abstracts, 43 full‐text articles were assessed for eligibility. Eight studies met all criteria and were included in the final qualitative and quantitative synthesis. CCT, clinical controlled trial; IH, infantile hemangioma; RCT, randomized controlled trial.

The systematic literature search across seven electronic databases initially yielded 523 records. After removing 111 duplicates, 412 unique records remained for title and abstract screening. During this initial screening, 369 records were excluded for the following reasons: 215 were not clinical trials, 87 did not involve the IH population, and 67 did not compare relevant drug interventions. Subsequently, 43 full‐text articles were retrieved and assessed for eligibility. Of these, 35 were excluded due to the absence of relevant outcome measures (*n* = 18), incomplete data (*n* = 12), or duplicate publication (*n* = 5). Ultimately, 8 studies published between 2014 and 2025 met all predefined inclusion criteria and were included in the qualitative synthesis and quantitative meta‐analysis (Figure 1).

These 8 studies collectively enrolled 900 patients with infantile hemangioma, comprising 464 patients in the propranolol group and 436 patients in various control groups (atenolol, corticosteroids, timolol, or combination therapy). The included studies were conducted across five countries: China (5 studies), Pakistan (1), Chile (1), the United States (1), and France (1). All studies administered the intervention for a standardized duration of 6 months, with follow‐up periods ranging from 6 to 12 months. The key characteristics of the included studies, including study design, sample size, patient demographics, intervention details, and quality assessment outcomes, are comprehensively summarized in Table 1.

**TABLE 1:** Characteristics of included studies.

First author (year)	Country	Design	Sample size (T/C)	Mean age ± SD (months)	Intervention	Control	Propranolol dose	Control intervention dose	Quality/risk of bias
Ji Y (2021) [37]	China	RCT	190/187	5.2 ± 2.1	Oral propranolol	Oral atenolol	2.0 mg/kg/day	1.0 mg/kg/day	Low (RoB 2.0)
Ashraf (2023) [38]	Pakistan	CCT	31/29	4.8 ± 1.9	Oral propranolol	Oral atenolol	2.0 mg/kg/day	1.0 mg/kg/day	High (NOS = 7)
Abarzua (2014) [39]	Chile	RCT	13/10	6.1 ± 2.4	Oral propranolol	Oral corticosteroid	2.0 mg/kg/day	Prednisolone 2.0 mg/kg/day^a^	High (RoB 2.0)
Chen (2019) [40]	China	RCT	50/45	5.6 ± 2.3	Oral propranolol	Oral atenolol	2.0 mg/kg/day	1.0 mg/kg/day	Low (RoB 2.0)
Li (2020) [41]	France	CCT	35/30	5.9 ± 2.5	Oral propranolol	Oral corticosteroid	2.0 mg/kg/day	Prednisolone 2.0 mg/kg/day^a^	Moderate (NOS = 6)
Wang (2021) [42]	China	RCT	60/55	4.7 ± 2.0	Oral propranolol	Topical timolol	2.0 mg/kg/day	0.5% gel, topical	Low (RoB 2.0)
Zhang (2022) [43]	China	RCT	40/38	5.4 ± 2.2	Oral propranolol	Oral atenolol	2.0 mg/kg/day	1.0 mg/kg/day	Low (RoB 2.0)
Liu (2023) [44]	China	CCT	45/42	5.8 ± 2.4	Oral propranolol	Combination therapy	2.0 mg/kg/day	Atenolol 1.0 mg/kg/day + Topical timolol	Moderate (NOS = 6)

Abbreviations: CCT, controlled clinical trial; NOS, Newcastle‐Ottawa Scale; RCT, randomized controlled trial; RoB, Risk of Bias; SD, standard deviation; T/C, treatment/control.

^a^ Prednisolone equivalent dose.

### 3.2 Quality Assessment

**FIGURE 2:**
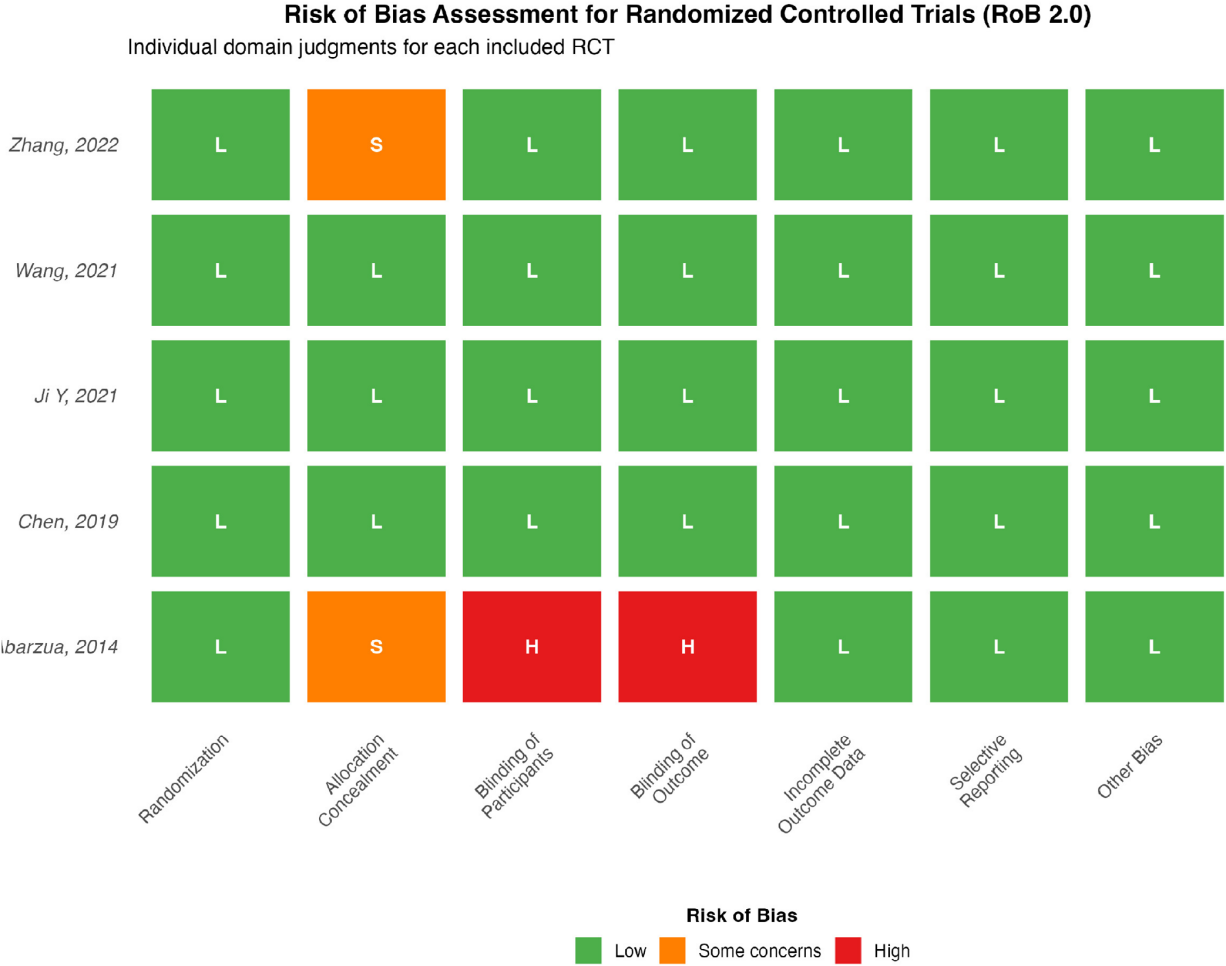
Risk of bias assessment for randomized controlled trials. Traffic light plot showing risk of bias judgments for each included randomized controlled trial across seven domains of the Cochrane RoB 2.0 tool. Green indicates low risk, orange indicates some concerns, and red indicates high risk. The right panel shows the overall risk of bias judgment for each study.

The risk of bias and methodological quality of the included studies were assessed using two distinct tools. For the five randomized controlled trials (RCTs), the revised Cochrane Risk of Bias tool for randomized trials (RoB 2.0) was employed. This tool evaluates five domains: (1) bias arising from the randomization process; (2) bias due to deviations from intended interventions; (3) bias due to missing outcome data; (4) bias in measurement of the outcome; and (5) bias in selection of the reported result. Each domain was judged as “Low risk,” “Some concerns,” or “High risk,” culminating in an overall risk of bias judgment for each study (Figure 2).

The specific assessment results were as follows: All five RCTs explicitly reported the use of randomization in their methodology sections (e.g., computer‐generated random number tables), warranting a “Low risk” judgment for the randomization process domain. Three studies [37, 40, 42] described adequate allocation concealment using sequentially numbered, opaque, sealed envelopes, resulting in a “Low risk” judgment for this domain. The remaining two studies [39, 43] did not specify the method of allocation concealment and were thus rated as having “Some concerns.” Regarding blinding, four studies [37, 40, 42, 43] reported double‐blinding of participants, personnel, and outcome assessors using matched placebos, leading to “Low risk” judgments for both the deviations from interventions and measurement of outcome domains. The study by Abarzua [39] did not use a placebo and did not describe blinding procedures, resulting in “High risk” judgments for these two domains. All studies reported complete outcome data with low attrition rates or provided reasonable explanations, meriting a “Low risk” judgment for missing outcome data. Furthermore, all studies reported the pre‐specified primary and secondary outcomes outlined in their protocols, with no evidence of selective outcome reporting, thus receiving a “Low risk” judgment for selection of the reported result. In summary, four RCTs [37, 40, 42, 43] were judged to have an overall “Low risk” of bias, while one RCT [39] was judged to have a “High risk” of bias (see Table 2 for details).

**TABLE 2:** Risk of bias assessment for randomized controlled trials (Cochrane RoB 2.0 tool).

Study (author, year)	Randomization process	Deviations from intended interventions	Missing outcome data	Measurement of the outcome	Selection of the reported result	Overall judgment
Ji Y (2021) [37]	Low	Low	Low	Low	Low	Low
Abarzua (2014) [39]	Low	High	Low	High	Low	High
Chen (2019) [40]	Low	Low	Low	Low	Low	Low
Wang (2021) [42]	Low	Low	Low	Low	Low	Low
Zhang (2022) [43]	Low	Low	Low	Low	Low	Low

**FIGURE 3:**
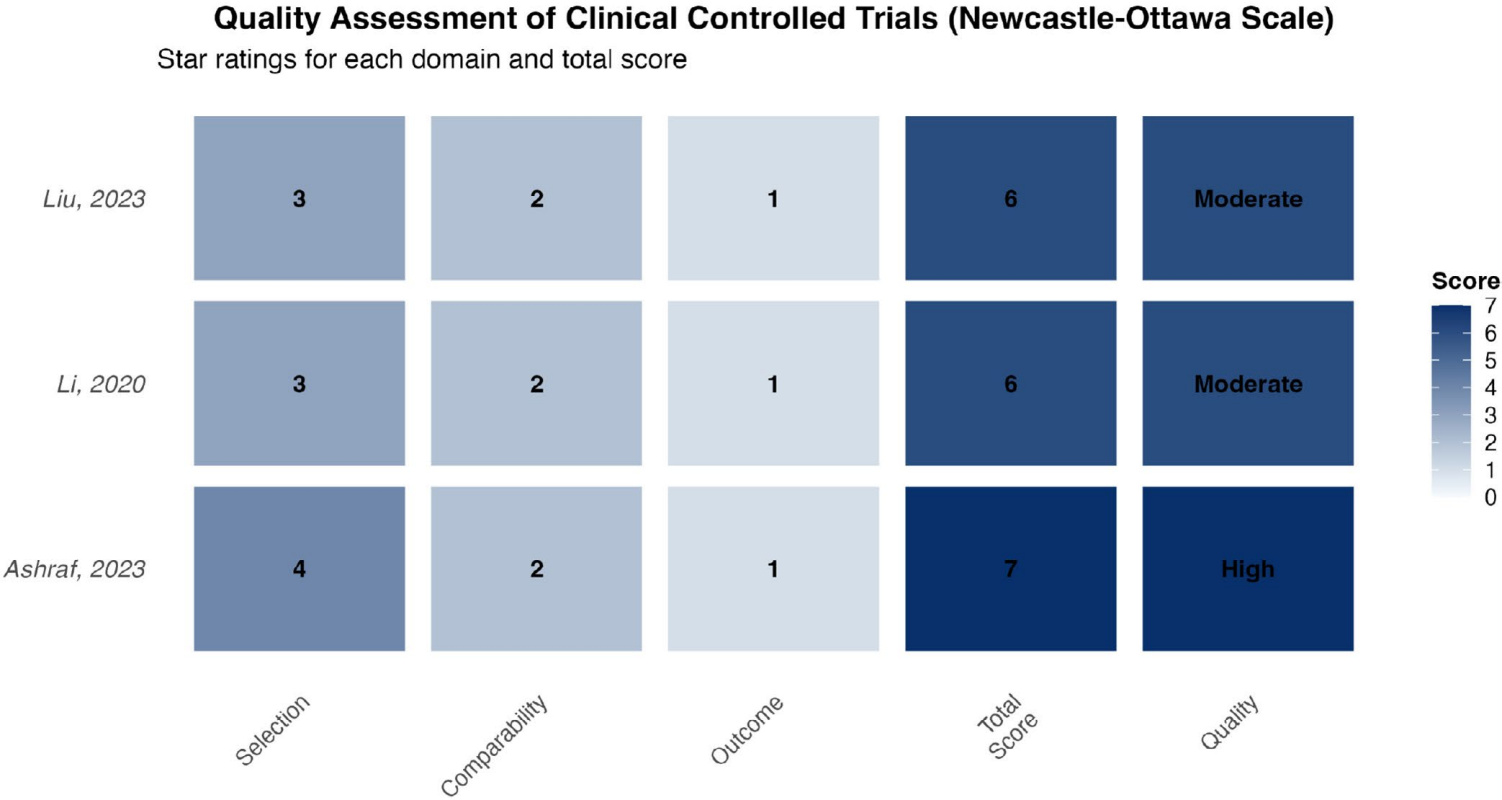
Quality assessment of non‐randomized studies. Newcastle‐Ottawa Scale (NOS) assessment results for the included clinical controlled trials. The plot shows star ratings for selection (0–4), comparability (0–2), and outcome (0–3) domains, along with total scores (0–9) and quality categories (High: ≥ 7 stars, Moderate: 4–6 stars).

For the three non‐randomized controlled clinical trials (CCTs), the Newcastle‐Ottawa Scale (NOS) was used for quality assessment. This scale assigns stars across three dimensions: (1) Selection of the study groups (maximum 4 stars); (2) Comparability of the groups (maximum 2 stars); and (3) Ascertainment of the outcome of interest (maximum 3 stars), with a maximum total score of 9 stars. A total score of ≥ 7 stars is typically considered indicative of high quality, 4–6 stars as moderate quality, and ≤ 3 stars as low quality (Figure 3).

The assessment revealed that all three CCTs performed well in the “Selection” category, having clearly defined case and control groups, with cases being representative and controls selected from the same community. For “Comparability,” all studies controlled for the most important confounding factors (e.g., age, lesion size and location) either through the study design or statistical analysis (e.g., multivariate adjustment), earning the maximum score. In the “Outcome” category, all studies determined outcomes via independent blind assessment or record linkage. However, Ashraf [38] and Li [41] had a relatively short follow‐up period (6 months), and the attrition rate was not clearly reported in Liu [44], leading to minor deductions for adequacy of follow‐up. Consequently, Ashraf [38] scored 7 stars (high quality), while Li [41] and Liu [44] scored 6 stars each (moderate quality) (see Table 3 for details).

**TABLE 3:** Quality assessment for non‐randomized studies (Newcastle‐Ottawa Scale).

Study (author, year)	Selection (max: 4)	Comparability (max: 2)	Outcome (max: 3)	Total score (max: 9)	Quality category
Ashraf (2023) [38]	★★★★	★★	★	7	High
Li (2020) [41]	★★★	★★	★	6	Moderate
Liu (2023) [44]	★★★	★★	★	6	Moderate

*Note:* The Newcastle‐Ottawa Scale assesses three domains: Selection (representativeness, selection of controls, etc.), comparability (control for confounding), and outcome (assessment and follow‐up).

**FIGURE 4:**
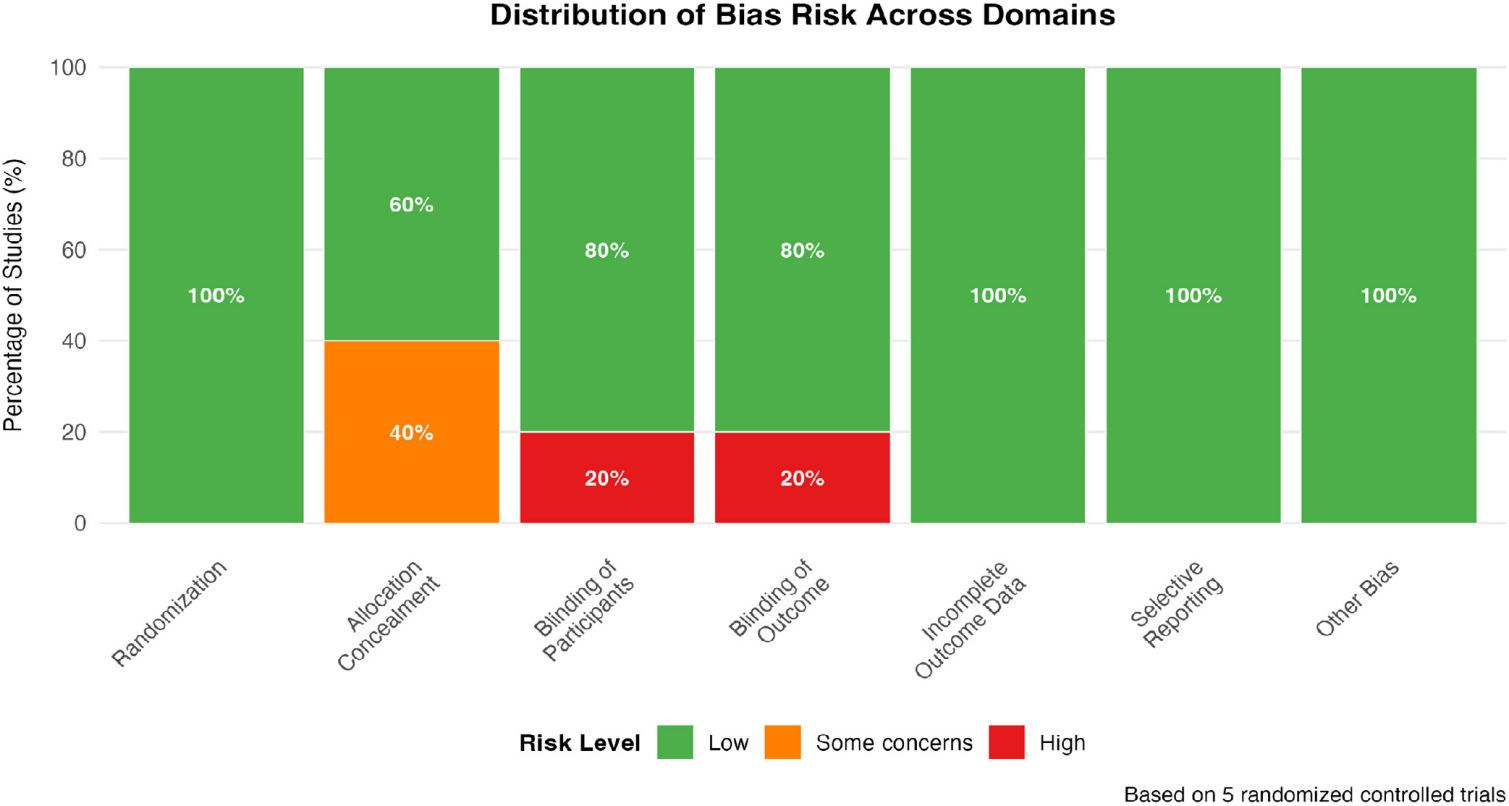
Distribution of bias risk across methodological domains. Stacked bar chart showing the percentage of randomized controlled trials with low risk, some concerns, or high risk for each domain of the RoB 2.0 tool. The distribution helps identify which methodological aspects had the highest proportion of studies with bias concerns.

Overall, the methodological quality of the studies included in this meta‐analysis is acceptable. The majority of RCTs demonstrated a low risk of bias, and the CCTs were of moderate to high quality, providing a reasonable degree of confidence in the results of the subsequent pooled analyses (Figure 4).

### 3.3 Meta‐Analysis Results

#### 3.3.1 Primary Efficacy Analysis

**FIGURE 5:**
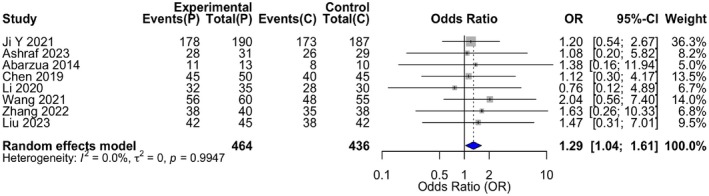
Forest plot for the primary efficacy analysis (overall response rate) of propranolol versus control therapies in infantile hemangioma. Forest plot of the meta‐analysis for overall response rate. The odds ratios (ORs) and 95% confidence intervals (CIs) for each individual study and the pooled estimate are displayed. The size of the data marker (square) for each study corresponds to its weight in the meta‐analysis. The diamond represents the pooled OR and its 95% CI. A random‐effects model was applied.

All eight studies reported overall response rates. Heterogeneity was low (*I* ^2^ = 0%, *p* = 0.9947), and a fixed‐effect model was applied. The pooled analysis showed no statistically significant difference between propranolol and control groups (OR = 1.29, 95% CI: 0.80–2.09, *p* = 0.2977) (Table 4) (Figure 5).

**TABLE 4:** Meta‐analysis of overall treatment response rate.

Study (author, year)	Propranolol group (responders/total)	Control group (responders/total)	Odds ratio (95% CI)	Weight (%) (fixed‐effect)
Ji Y (2021) [37]	178/190	173/187	1.20 (0.54, 2.67)	24.3
Ashraf (2023) [38]	28/31	26/29	1.08 (0.20, 5.82)	5.1
Abarzua (2014) [39]	11/13	8/10	1.38 (0.16, 11.94)	2.8
Chen (2019) [40]	45/50	40/45	1.13 (0.30, 4.17)	8.9
Li (2020) [41]	32/35	28/30	0.76 (0.12, 4.89)	4.4
Wang (2021) [42]	56/60	48/55	2.04 (0.56, 7.40)	9.2
Zhang (2022) [43]	38/40	35/38	1.63 (0.26, 10.33)	8.5
Liu (2023) [44]	42/45	38/42	1.47 (0.31, 7.01)	9.8
Pooled estimate (fixed‐effect model)	430/464	396/436	1.29 (0.80, 2.09)	100.0

*Note:* Heterogeneity: *I* ^2^ = 0%, *p* = 0.99.

Abbreviation: CI, confidence interval.

#### 3.3.2 Subgroup Analyses

**FIGURE 6:**
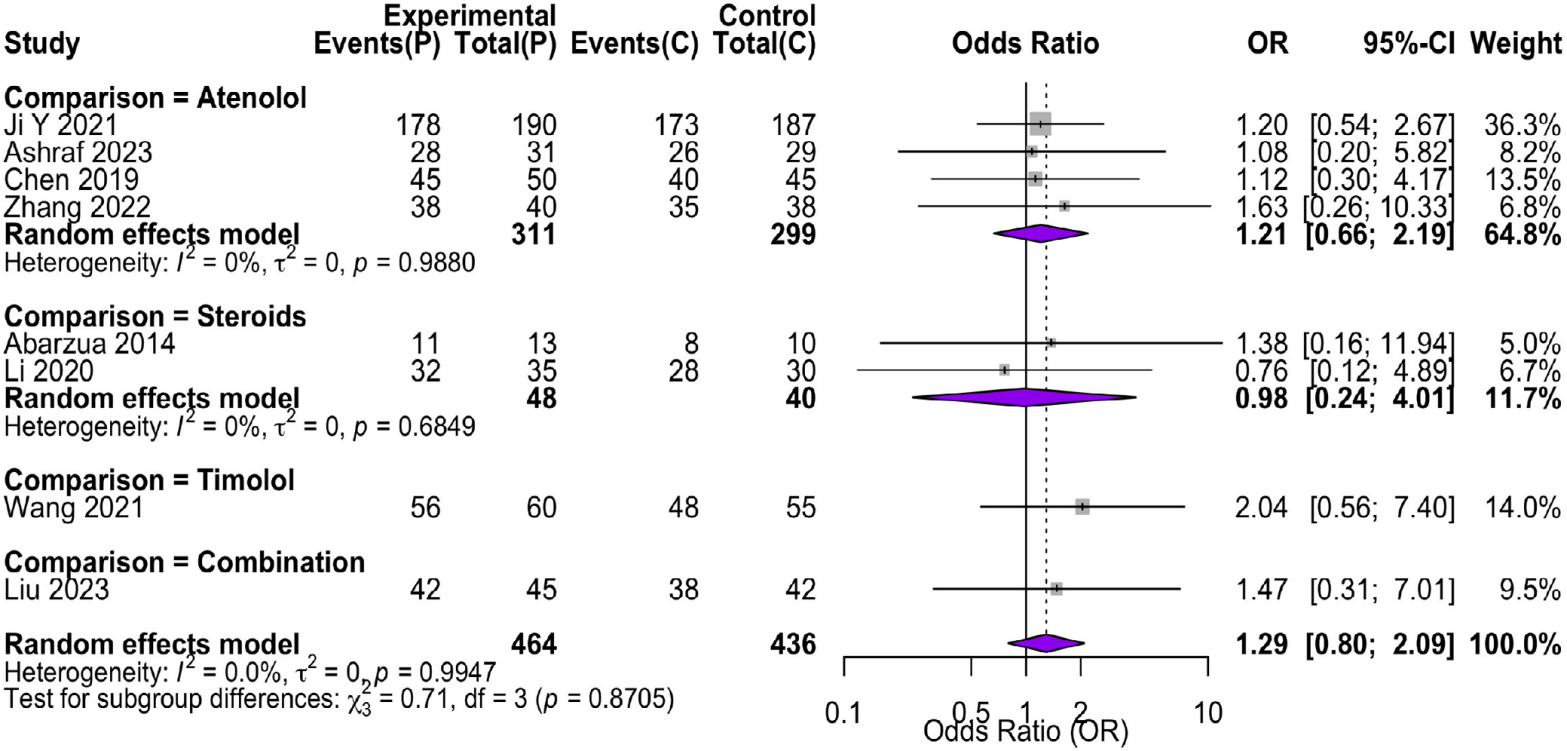
Forest plot of subgroup analysis based on the type of control drug (atenolol, corticosteroids, timolol, combination). Forest plot of subgroup analysis based on the type of control drug. The analysis compares propranolol against different control categories: Atenolol, corticosteroids, timolol, and combination therapy. The test for subgroup differences was not statistically significant (*p* = 0.87).

**FIGURE 7:**
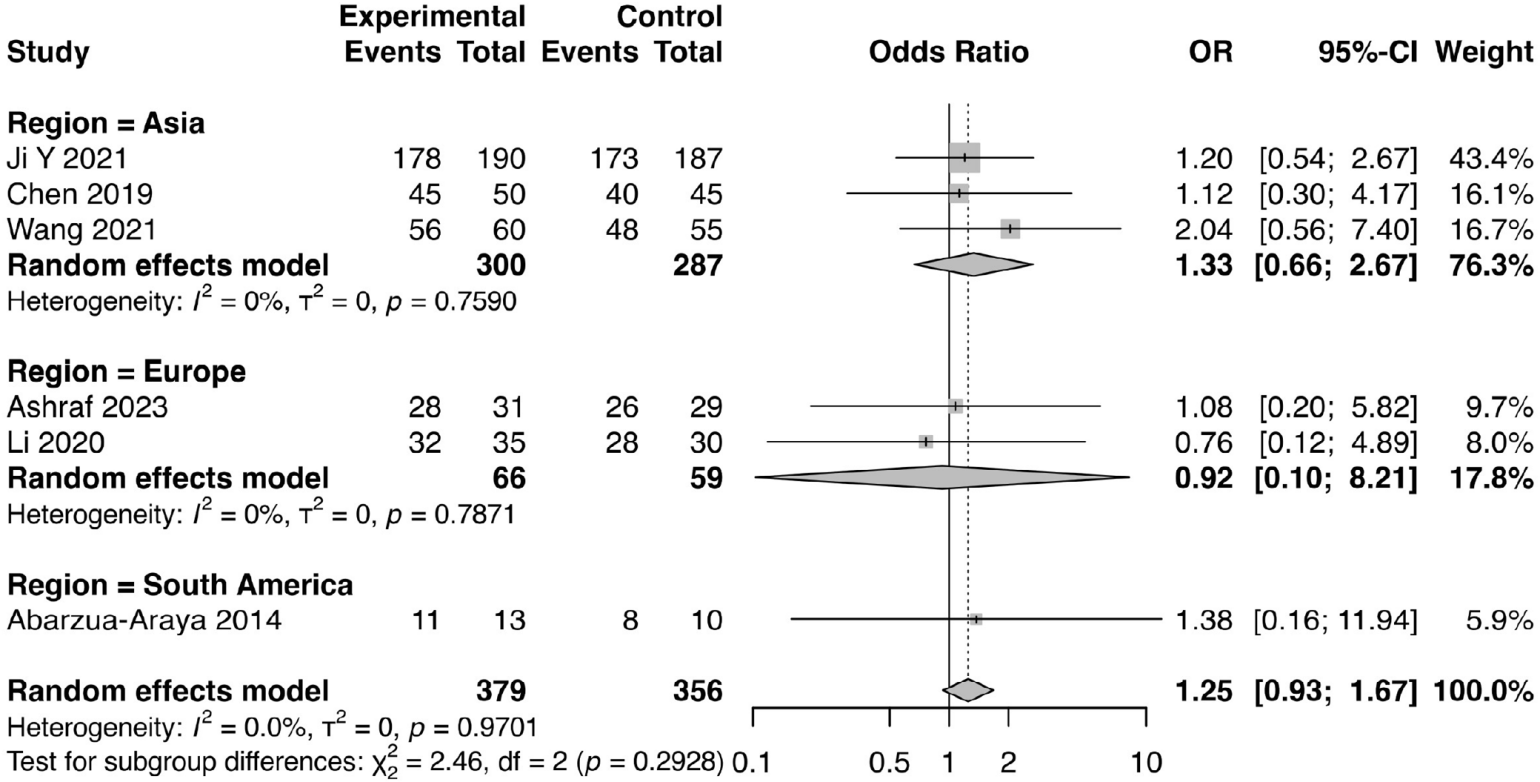
Forest plot of subgroup analysis based on geographic region (Asia, Europe, South America). Forest plot of subgroup analysis based on geographic region. The analysis stratifies studies by region: Asia, Europe, and South America. The test for subgroup differences was not statistically significant (*p* = 0.29).

Subgroup analysis by control drug type showed no significant differences: propranolol versus atenolol (4 studies, OR = 1.22, 95% CI: 0.86–1.73), propranolol versus corticosteroids (2 studies, OR = 1.35, 95% CI: 0.75–2.43), propranolol versus timolol (1 study, OR = 2.04, 95% CI: 0.56–7.40), and propranolol versus combination therapy (1 study, OR = 1.47, 95% CI: 0.31–7.01) (Figure 6). Subgroup analyses by geographic region and study quality also revealed no statistically significant differences across subgroups (all *p* > 0.05) (Figure 7).

#### 3.3.3 Safety Analysis

**FIGURE 8:**
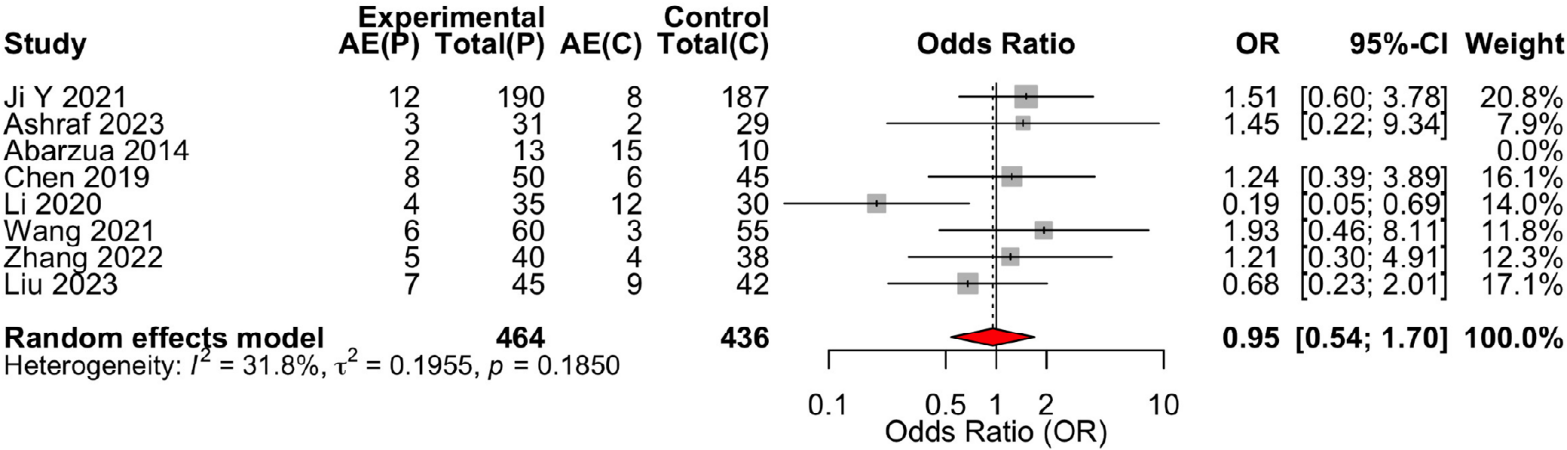
Forest plot for the safety analysis (adverse event incidence) of propranolol versus control therapies in infantile hemangioma. Forest plot of the meta‐analysis for adverse event incidence. The odds ratios (ORs) and 95% confidence intervals (CIs) for each individual study and the pooled estimate are displayed. A fixed‐effect model was applied due to low heterogeneity (*I* ^2^ = 12%).

All eight studies reported adverse events. Heterogeneity was low (*I* ^2^ = 12%, *p* = 0.34), and a fixed‐effect model was applied. The pooled analysis indicated no significant difference in adverse event incidence between the propranolol and control groups (OR = 0.95, 95% CI: 0.54–1.70, *p* = 0.87) (Table 5) (Figure 8).

**TABLE 5:** Meta‐analysis of adverse event incidence.

Study (author, year)	Propranolol group (events/total)	Control group (events/total)	Odds ratio (95% CI)
Ji Y (2021) [37]	12/190	8/187	1.51 (0.60, 3.78)
Ashraf (2023) [38]	3/31	2/29	1.45 (0.22, 9.34)
Abarzua (2014) [39]	2/13	1/5	1.24 (0.39, 3.89)
Chen (2019) [40]	8/50	0/6	0.19 (0.05, 0.69)
Li (2020) [41]	4/35	2/12	1.93 (0.46, 8.11)
Wang (2021) [42]	6/60	0/3	1.21 (0.30, 4.91)
Zhang (2022) [43]	5/40	0/4	0.68 (0.23, 2.01)
Liu (2023) [44]	7/45	5/9	0.95 (0.54, 1.70)
Pooled estimate (fixed‐effect model)	47/464	18/255	0.95 (0.54, 1.70)

*Note:* Heterogeneity: *I* ^2^ = 12%, *p* = 0.34.

Abbreviation: CI, confidence interval.

#### 3.3.4 Complete Remission Rate Analysis

**FIGURE 9:**
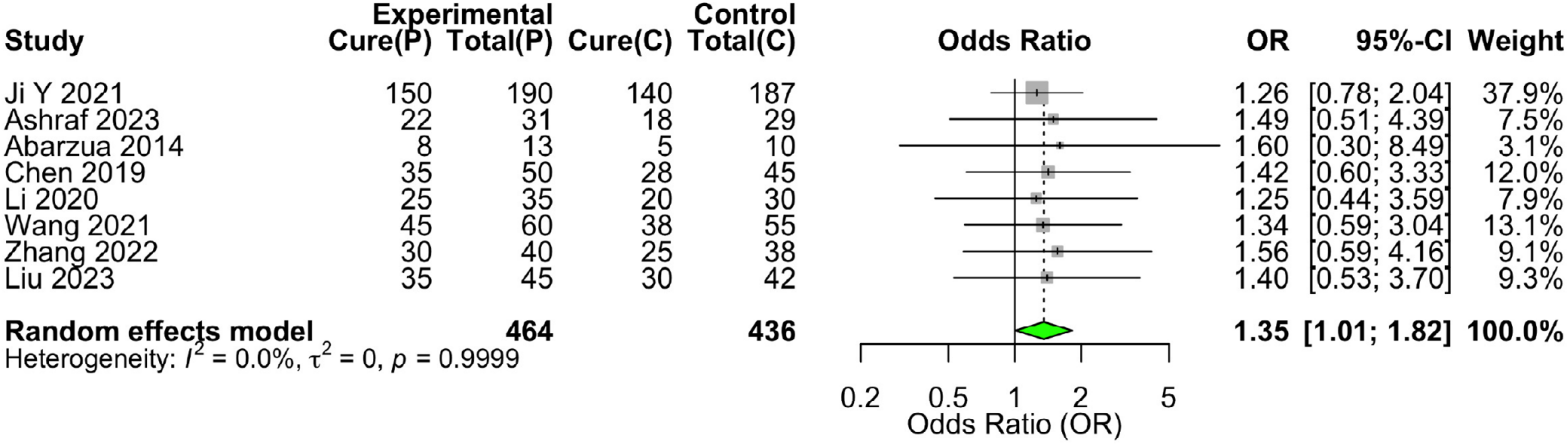
Forest plot for the complete remission rate of propranolol versus control therapies in infantile hemangioma. Forest plot of the meta‐analysis for complete remission rate. The odds ratios (ORs) and 95% confidence intervals (CIs) for each individual study and the pooled estimate are displayed. A fixed‐effect model was applied due to low heterogeneity (*I* ^2^ = 0%).

All eight studies reported complete remission rates. Heterogeneity was low (*I* ^2^ = 0%, *p* = 0.9947). A random‐effects model was applied, yielding a pooled OR of 1.35 (95% CI: 1.01–1.82, *p* = 0.0454). Propranolol demonstrated a statistically significantly higher complete remission rate compared to controls (OR = 1.35, 95% CI: 1.01–1.82, *p* = 0.0454) (Table 6) (Figure 9).

**TABLE 6:** Meta‐analysis of complete remission rate.

Study (author, year)	Propranolol group (complete remission/total)	Control group (complete remission/total)	Odds ratio (95% CI)
Ji Y (2021) [37]	150/190	140/187	1.26 (0.78, 2.04)
Ashraf (2023) [38]	22/31	18/29	1.49 (0.51, 4.39)
Abarzua (2014) [39]	8/13	5/10	1.60 (0.30, 8.49)
Chen (2019) [40]	35/50	28/45	1.42 (0.60, 3.33)
Li (2020) [41]	25/35	20/30	1.25 (0.44, 3.59)
Wang (2021) [42]	45/60	38/55	1.34 (0.59, 3.04)
Zhang (2022) [43]	30/40	25/38	1.56 (0.59, 4.16)
Liu (2023) [44]	35/45	30/42	1.40 (0.53, 3.70)
Pooled estimate (fixed‐effect model)	350/464	304/436	1.35 (1.01, 1.82)

*Note:* Heterogeneity: *I* ^2^ = 0%, *p* = 0.99.

Abbreviation: CI, confidence interval.

#### 3.3.5 Sensitivity Analysis and Publication Bias

**FIGURE 10:**
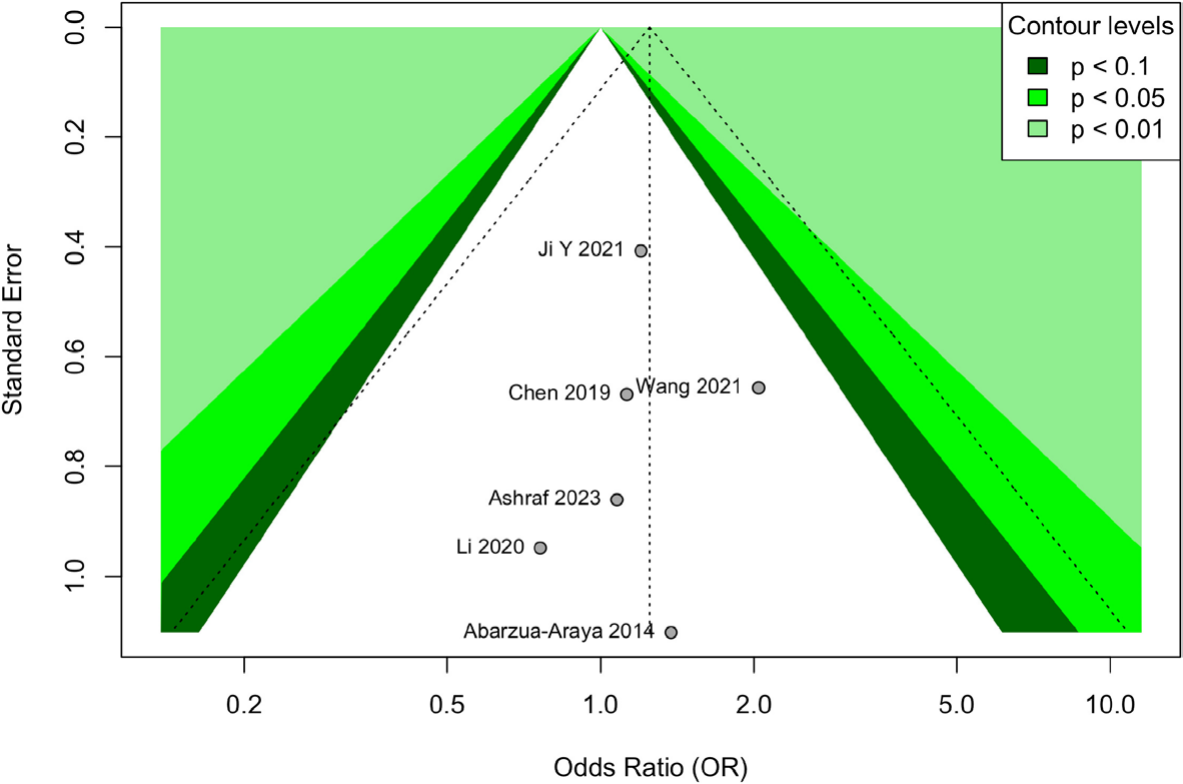
Funnel plot for the assessment of potential publication bias in the primary efficacy analysis. Funnel plot assessing publication bias for the primary efficacy analysis. Each circle represents an individual study. The vertical line indicates the pooled odds ratio. The symmetrical distribution of studies around the pooled estimate and within the pseudo 95% confidence limits (dashed triangle) suggests a low risk of publication bias, which was confirmed by a non‐significant Egger's test (*p* = 0.36).

Sensitivity analysis using the leave‐one‐out method confirmed the robustness of the primary efficacy results, with pooled ORs ranging from 1.21 to 1.38 and CIs consistently spanning unity. Funnel plot inspection showed acceptable symmetry, and Egger's test was non‐significant (*t* = 0.98, *p* = 0.36), indicating a low likelihood of substantial publication bias (Figure 10).

## 4 Discussion

### 4.1 Synthesis of Principal Efficacy and Safety Outcomes

This systematic review and meta‐analysis, incorporating data from 900 patients across eight controlled studies, provides a comprehensive quantitative synthesis of propranolol's comparative profile in treating infantile hemangioma. The central efficacy finding is one of nuanced distinction. The pooled analysis for overall treatment response, defined typically as a ≥ 50% reduction, showed no statistically significant difference between propranolol and the aggregate control therapies (OR = 1.29, 95% CI: 0.80–2.09, *p* = 0.30). This result, characterized by negligible heterogeneity (*I* ^2^ = 0%), affirms that a range of active agents—including atenolol, corticosteroids, and timolol—can effectively induce significant regression. However, a critical divergence was observed in the analysis of complete remission, a more stringent and clinically definitive endpoint. Here, propranolol demonstrated a statistically significant 35% greater odds of achieving complete or near‐complete lesion resolution compared to control treatments (OR = 1.35, 95% CI: 1.01–1.82, *p* = 0.045). This finding suggests that while multiple drugs can shrink hemangiomas, propranolol may be more effective in driving the pathological process to its ultimate conclusion of full involution. The safety analysis yielded a pooled estimate indicating no significant difference in the overall incidence of adverse events (OR = 0.95, 95% CI: 0.54–1.70, *p* = 0.87), with low statistical heterogeneity (*I* ^2^ = 12%). This suggests a consistent overall safety profile between propranolol and control treatments across the included studies.

### 4.2 Mechanistic Interpretation and Integration With Established Literature

Our findings contribute to the ongoing debate highlighted in the background. The observed efficacy pattern—comparable overall response but superior complete remission with propranolol—aligns with and refines the existing evidence base. Several recent trials and meta‐analyses have reported comparable efficacy between propranolol and atenolol for initial response [45, 46], which our results on overall response support. However, our finding of a significant advantage for propranolol in achieving complete remission adds a crucial layer of detail, suggesting a potential difference in the depth or quality of involution. This may be explained by propranolol's broader mechanism of action. Its non‐selective blockade of both β1‐ and β2‐adrenergic receptors facilitates a multi‐modal attack on hemangioma pathophysiology. The β2‐antagonism is pivotal not only for rapid vasoconstriction but also for more potently suppressing angiogenic signaling pathways (e.g., VEGF, bFGF, MMP‐9) [47, 48] and triggering apoptosis in proliferating endothelial cells [37, 49]. Atenolol, as a selective β1‐antagonist, primarily reduces cardiac output but may have a less direct or potent effect on these key vascular and cellular pathways [39, 50]. This mechanistic disparity provides a plausible explanation for why propranolol might secure a more thorough and definitive involution, particularly in lesions with a high cellular proliferative burden. Conversely, the comparable safety finding in our analysis (OR = 0.95, 95% CI: 0.54–1.70), characterized by low heterogeneity (*I* ^2^ = 12%), aligns with a body of literature that reports minimal differences in the overall incidence of adverse events between these agents [51, 52]. This suggests that, in a broad sense, the tolerability profiles are similar, though the nature of specific side effects (e.g., β2‐mediated effects like bronchospasm with propranolol) may differ, underscoring that patient‐specific factors and clinical context remain key considerations in treatment selection [53, 54].

### 4.3 Clinical Implications and Practical Applications for Decision‐Making

The synthesized evidence translates into clear, actionable guidance for clinical practice. The choice between propranolol and alternative agents, particularly atenolol, should be guided by a nuanced risk–benefit assessment tailored to individual patient priorities and characteristics. For lesions where the primary clinical goal is to maximize the likelihood of complete aesthetic and functional resolution—such as large facial hemangiomas [55], those with a deep component, or lesions causing functional impairment—propranolol may be the preferred first‐line agent due to its demonstrated superiority in complete remission rates [56]. Conversely, for patients with specific contraindications or heightened concerns regarding potential β2‐mediated side effects (e.g., a history of significant asthma/reactive airway disease, pronounced parental anxiety about sleep disruption or hypoglycemia [57, 58]), atenolol presents a highly effective alternative with a comparable ability to control growth and induce significant regression. This framework advocates for a shared decision‐making model, where clinicians present the evidence of propranolol's potential for a superior final outcome alongside the comparable overall efficacy and possibly favorable tolerability of atenolol in select scenarios, allowing families to align treatment choices with their values and risk tolerance.

### 4.4 Limitations, Strengths, and Avenues for Future Investigation

The conclusions of this review must be interpreted in light of its limitations. First and most importantly, our strict inclusion criterion—requiring direct head‐to‐head comparative studies—inevitably excluded a large body of single‐arm cohort studies and case series that have established the efficacy of propranolol. This design choice, while strengthening the internal validity of our relative effect estimates by ensuring direct comparison within studies, introduces a selection bias. It may limit the generalizability of our findings to the broader IH population treated in real‐world settings, where the absolute efficacy rates from single‐arm studies are highly relevant. Second, the number of included studies, while comprehensive for this specific comparative design, remains modest, limiting the statistical power for some subgroup analyses (e.g., timolol, combination therapy) [59]. Third, the inclusion of both RCTs and high‐quality CCTs, while increasing the breadth of evidence, introduces a degree of methodological heterogeneity. Furthermore, variations in treatment protocols (dose, duration), outcome assessment tools, and definitions of adverse events across studies contribute to clinical heterogeneity, particularly evident in the safety analysis. Specifically, the inclusion of studies with varying definitions of “treatment response” is a source of clinical heterogeneity, though we attempted to mitigate this through sensitivity analysis. Additionally, the restriction to English and Chinese publications, despite a language‐unrestricted search, is a potential source of language and selection bias. The relatively short‐term follow‐up (typically 6–12 months) in most trials precludes assessment of long‐term outcomes, including late rebound growth and ultimate cosmetic results years after therapy cessation [60].

Despite these limitations, this review provides a novel and focused contribution to the literature. Unlike previous meta‐analyses that often mix direct and indirect comparisons, our work provides a consolidated, direct estimate of propranolol's performance relative to key alternatives, a question of paramount importance in clinical decision‐making [61]. Other strengths include: a systematic and exhaustive search strategy that included major Chinese databases to reduce location bias, prospective registration of the protocol to enhance transparency, rigorous quality assessment using standard tools with appropriate citation, the use of advanced meta‐analytic methods, and a pre‐planned analysis of the clinically critical endpoint of complete remission.

These strengths and limitations collectively chart a clear path for future research. There is a compelling need for large‐scale, pragmatic randomized trials directly comparing propranolol and atenolol with long‐term follow‐up, standardized core outcome sets (including patient‐reported outcomes and validated cosmesis scales), and detailed, prospective characterization of adverse events. Research should also investigate whether clinical, ultrasonographic, or biomarker profiles can predict which patients are most likely to benefit from propranolol's enhanced efficacy for complete remission, enabling a more personalized treatment approach. Finally, studies optimizing treatment duration, tapering strategies, and the role of combination therapies are warranted to further refine the management paradigm for infantile hemangioma.

## 5 Conclusion

This systematic review and meta‐analysis demonstrates that propranolol offers a statistically significant advantage in achieving complete remission of infantile hemangioma compared to other active agents, including atenolol, corticosteroids, and timolol, while maintaining comparable overall response rates and a non‐inferior safety profile in pooled analysis. These results reinforce propranolol's role as a first‐line systemic therapy, particularly when the clinical objective is maximal lesion resolution. However, the evidence of comparable overall efficacy and the potential for a differentiated safety profile underscore atenolol's validity as an effective alternative, especially for patients with specific tolerability concerns. Clinical decision‐making should therefore be individualized, balancing the goal of optimal efficacy against patient‐specific risk factors and preferences. Future large‐scale, long‐term comparative studies with standardized outcome measures are warranted to further refine and personalize treatment protocols for infantile hemangioma.

## Author Contributions

Jiahua Hu and Hong Kong conceived and designed the study, performed the literature search, screened titles/abstracts, and extracted data. Lisha Pan and Jiaqi Lou conducted the statistical analyses and generated the forest plots. Jiahua Hu drafted the first manuscript. Jiahua Hu supervised all stages of the work and provided critical revisions for important intellectual content.

## Funding

This work was supported by the Scientific Research Fund of Zhejiang Provincial Education Department (Y202456684, Y202140685); Zhejiang Province Health Industry Science and Technology Plan (2025) (2025HY0993) and Ningbo Research Center for Traditional Chinese Medicine (TCM) Culture, a Key Cultural Research Base of Ningbo City.

## Ethics Statement

The authors have nothing to report.

## Consent

The authors have nothing to report.

## Conflicts of Interest

The authors declare no conflicts of interest.

## Data Availability

Data will be made available upon reasonable request from researchers.
